# Predictive ability of malnutrition screening tools in enterally fed, mechanically ventilated patients with phase angle inference: A prospective observational study

**DOI:** 10.62838/jccm-2026-0010

**Published:** 2026-04-30

**Authors:** Mahmoud Al-Kalaldeh, Mohammad Abu Sabra, Omar Al-Kalaldeh

**Affiliations:** Faculty of Nursing, The University of Jordan-Aqaba Campus, Aqaba, Jordan; Ministry of Health,Jordan

**Keywords:** ICU, malnutrition, phase angle, prediction, screening tools

## Abstract

**Background:**

The prognostic abilities of malnutrition assessment tools for the critically ill are still controversial. This study aimed to assess the predictive ability of MUST, NRS-2002, and NUTRIC tools to predict malnutrition risk for enterally fed, mechanically ventilated patients in intensive care.

**Methods:**

In a multicenter, prospective, observational study, patients from five ICU units in Jordan were observed at two stages. During the first 24 hours of admission, MUST, NUTRIC, and NRS-2002 scores were obtained in addition to the demographic and admission characteristics. In the assessment stage, on day 6th of admission forward, the Bioelectrical Impedance Analysis (BIA), including body compositions and Phase Angle (PhA) were assessed. Machine Learning (ML), structural equation modeling (SEM), and area under the curve (AUC) were used for measuring malnutrition estimates.

**Results:**

A total of 709 patients were observed. At admission, NUTRIC, MUST, and NRS-2002 were congruent in identifying high malnutrition risk (45.1%, 46.4%, and 53.6%, respectively). In reference to PhA, MUST and NRS-2002 scored higher for sensitivity (77.6%, and 76.8%, respectively) and specificity (93.3%, and 90.7%, respectively). They reported acceptable correlation estimates by SEM (0.65, and 0.70, respectively), and ML (0.90, and 0.91, respectively). Further, MUST was the best to discriminate malnutrition, followed by NRS-2002 and NUTRIC (AUC= 0.76, 0.64, and 0.53, respectively).

**Conclusions:**

Alongside validated BIA technology, MUST and NRS-2002 functioned as reliable prognostic indicators of malnutrition risk in the ICU.

## Introduction

Malnutrition is an increasingly challenging and prevalent issue in intensive care and its association between caloric delivery and caloric needs is emphasized [1, 2]. It is known that critically ill patients are at risk for malnutrition due to hypermetabolism, gut dysfunction, and poor prognosis [3, 4]. Malnutrition is the main reason for immune depletion, delayed recovery, prolonged stay in the intensive care unit (ICU), and increased mortality [5, 6]. According to the ESPEN, malnutrition is defined as “a state resulting from lack of intake or uptake of nutrition that leads to altered body composition (decreased fat-free mass) and body cell mass leading to diminished physical and mental function and impaired clinical outcome from disease” [2]. Being on mechanical ventilation (MV) is particularly associated with poorer prognosis, along with challenges to achieve successful weaning, especially after prolonged MV use [7, 8]. Despite its physiological superiority over parenteral nutrition, enteral nutrition (EN) is still connected with a range of feeding-related complications. These include, but are not limited to, aspiration, pneumonia, feeding intolerance, re-feeding syndrome, diarrhoea, and hyperglycaemia [9, 10]. The association between feeding-related complications and inadequate caloric attainment and malnutrition is well established [11, 12].

Focused assessment aims to maintain ongoing monitoring for certain nutritional outcomes to promote nutritional adequacy and early detection of malnutrition even when EN is initiated [13, 14]. It is seen that only 20−50 % of patients in the ICU receive the estimated energy requirement and have longer ICU stays [15, 16]. Clinical trials showed a significant reduction in serious clinical outcomes and mortality when malnutrition risk is determined early after admission [17]. Once nutritional risk is identified, a thorough nutritional assessment is required to quantify the severity of malnutrition [15].

In the absence of evidence-based tools for malnutrition screening in the critically ill, the early detection of potential or manifested cases of malnutrition is less likely [13, 12]. These tools should be validated, feasible, sensitive, and specific to assess patients in a timely manner. The early anticipation of malnutrition requires screening in the first 24 to 4 8 h after admission and in case of high risk, a repeated assessment a regular intervals is needed [18]. The majority of nutritional societies (i.e. ESPEN, ASPEN) validated the use of the malnutrition universal screening tool (MUST), Nutritional Risk Screening 2002 (NRS-2002), and Nutrition in the Critical Care (NUTRIC) score for nutritional screening including ICU and other hospitalized patients [19, 14, 20]. Assessment of body composition is more objective and accurate to estimate malnutrition based on describing body compartments such as muscle mass, fat mass, and water rations [21, 22]. Bioelectrical impedance analysis (BIA) technology is one of these validated methods that measure the previous body compartments in addition to the phase angle (PhA) by obtaining the resistance and reactance [23, 24].

This study aimed to assess the predictive ability of MUST, NUTRIC, and NRS-2002 tools to predict malnutrition risk for enterally fed, mechanically ventilated patients in intensive care in reference to PhA value. Various statistical procedures were used to achieve this objective, including the Machine Learning (ML) approach.

## Methods

This is a multicenter, prospective, longitudinal, observational study that assessed critically ill patients’ nutritional outcomes using different malnutrition screening tools at patients’ admission, and the BIA technology during patients’ stay in the intensive care unit (ICU). This study was conducted in five intensive care units located in three referral hospitals affiliated with different healthcare sectors in Jordan; the military royal medical services, the Ministry of Health, and the private sector hospital. The ethical research committee at the University of Jordan granted ethical approval to commence the study on 8th Sep 2021. Strengthening the Reporting of Observational Studies in Epidemiology (STROBE) guidelines were followed. The study was conducted in the period between September 2021 and May 2023.

### Sampling

Critically ill patients admitted to the ICU with different medical disorders and traumas were approached in this study. Of these admissions, patients who required mechanical ventilation (MV), whether emergently or electively, and enteral nutrition (EN) were selected for observation. These patients selected for bedside observation were adults aged between 20 – 65. Observation included only the newly admitted patients who had not already started any nutrition therapy. Therefore, patients who had already started on nutrition therapy before the data collection period were excluded even though they were eligible for participation. This is because the aim of the study was to commence observation from starting the nutritional therapy.

Patients known with some comorbidities such as severe heart failure (class IV), chronic kidney disease, chronic liver disorders, and immunocompromised patients were excluded from selection. In addition, patients with severe gastrointestinal disorders that contradict commencing EN therapy, and patients on parenteral nutrition were also excluded. In addition, patients with severe hemodynamically deteriorated conditions, which is defined as refractory hypotension that requires high doses of Inotropes such as >25μg/kg/min for Dopamine and Dobutamine, >0.5 μg/kg/min for Epinephrine and Norepinephrine, or >0.04 units/min for Vasopressin were also excluded.

The sample size was estimated using the following formula: n= z 2pq /d 2.

[n: sample size; z: Z-score of standard deviation which is equal to 1.96; p: proportion; q: 1-p; d: α=0.05] [**25**].

Based on the global prevalence of malnutrition among critically ill patients between 60−65% [13, 1, 4], the minimal size required for this study was 350 subjects. However, assuming various etiologies among the prospective patients that may produce heterogeneous effects, the sample size was doubled to reduce this threat.

### Bedside observation

At the admission stage, admission criteria were reported for each selected case and obtained only at the patient’s admission. These included the admission diagnosis (e.g. cardiovascular, respiratory, neurological… etc.), the acute physiology and chronic health evaluation II score (APACHE II), which is a universal tool for estimating the severity and mortality of acute illness, the sequential organ failure assessment (SOFA), and the body mass index (BMI). Alternative scales such as length of the forearm (ulna) and the height of knees to measure height, and mid-upper arm circumference were used to estimate BMI when ordinary height and weight measurements were no longer possible.

In addition to the previous parameters, the following are nutritional screening tools obtained once at the first 24 hours of admission. These include the Malnutrition Universal Screening Tool (MUST), Nutritional Risk Screening 2002 (NRS-2002), and the Nutrition in the Critical Care tool (NUTRIC). MUST was used to estimate the risk of malnutrition through measuring anthropometric characteristics such as height, weight, BMI, and weight change over time. MUST scores provide inferences for malnutrition at three levels; 0- Low risk, 1 - Medium risk, and 2+ - High risk [26]. The NUTRIC estimates the probability of critically ill patients responding to aggressive nutritional therapy. The score consists of different parameters such as age, APACHE II score, SOFA score, number of co-morbidities, and length of stay in the ICU [27]. Patients who scored between 0−4 NUTRIC scores are considered at low nutritional risk, whereas patients who scored between 5−9 are considered at high nutritional risk, considering the absence of an Interleukin-6 score (IL-6) [20]. The NRS-2002 can also detect the likelihood of benefit from nutritional therapy in hospitalized patients [15]. The NRS-2002 is a well-validated tool that encompasses the pre-screening of four questions. If one of these questions is positive, a subsequent screening including different measures of nutritional assessment, considering the severity of the disease, was followed. Each parameter can be scored from 0 to 3. A total NRS-2002 score of ≥3 points indicates that the patient has a high risk of malnutrition and requires intensive nutritional therapy [19].

The assessment stage of nutritional assessment was conducted after the day 6^th^ of admission (preferably between the day 6^th^ and day 14^th^ of ICU admission), using the bioelectrical impedance analysis (BIA). ‘InBody S10’, produced by (Biospace, Seoul, Korea), is a recommended BIA device known for its reliability and precision through 8 electrodes attached to a patient’s hand, anklebones, and heels [23]. This technique was used to measure the segmental body compositions objectively by analyzing the resistance and reactance of tissue when a current frequency of 50 kHz pass through it. Different values can be obtained from the BIA including body fat mass, skeletal muscle mass (SMM), extracellular water/total body water (ECW/TBW) ratio, and the phase angle (PhA). PhA value was articulated from the raw data retrieved from BIA results, which were used for malnutrition prognosis [21, 28].

While PhA is increasingly used in clinical practice, its estimated cutoff value is still not consolidated to incorporate its inference with other nutritional risk screening methods, especially for the first 72 hours of admission [29, 30]. However, pre-defined PhA values were introduced differentially between studies. For instance, the lowest median PhA was observed in a study of 4.4º [31] and another study of 3.9 [32]. Another study reported a higher PhA mean at 5.1º [33]. Thus, a PhA cutoff point between 3−5 was introduced in a number of studies to predict the poorest prognosis for longer ICU stays, considering a sensitivity value >90% and specificity >65% [31, 33, 34]. Based on these presuppositions, the cutoff value of PhA in this study was set at 4º. Figure 1 shows the flow of the study observation tools.

**Figure:**
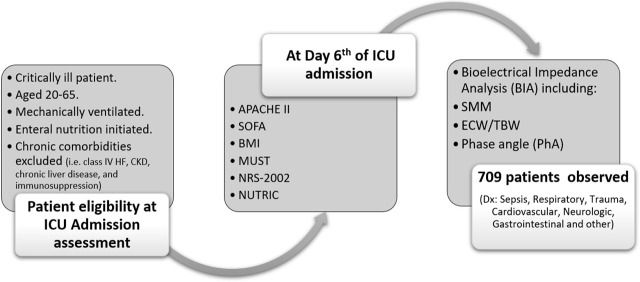


### Data collection

Bedside observation was conducted by the researcher and other research assistants working in the assigned ICUs. Arrangements between the researchers and the ICUs’ head/in-charge nurses were anticipated to facilitate identifying eligible patients who were admitted to these units and required MV during the early 24−48 hours from admission. The introductory session was provided for each unit staff to enhance familiarity with the study objectives and requirements. Once a patient meeting the inclusion criteria was admitted to the unit, the researcher was contacted on the same day or the day next to commence gathering information about the patient at the admission stage. A special form was provided for all research teams to document all data in scheduled observation dues, considering the initiation of EN therapy. It was the responsibility of the researchers to keep in contact with all research assistants to report observation parameters precisely. To attempt to validate the observation data, the principal investigator (MK) performed random inter-observer checks for some of the recorded data. Regular visits for surveillance were ensured, including direct follow-ups for unexpected events such as terminating observation due to medical deterioration, patients’ transfer, discharge, or death. The overall study procedure was reviewed by a consultant intensivist and registered dietitian who validated the study observation protocol and provided suggestions prior to data collection.

### Statistical analysis

Data was entered into the SPSS software (IBM Corp. IBM SPSS Statistics for Windows, Version 26. Armonk, NY). Before initiating data analysis, checking for missing and outliers was considered and managed by returning to the original observation sheets. Descriptive statistics used for data included frequency, percentage, mean, standard deviation (SD), median, interquartile range (IQR), and range. Cross tabulation was used to examine sensitivity, specificity, positive predictive value (PPV), negative predictive value (NPV), and accuracy for all assessed screening tools in congruence with PA value. The relationship between significantly correlated variables (factors) and PA was also assessed using a structural equation model, which was developed using the SPSS Amos software (IBM, SPSS, Amos, version 26). This multivariate statistical analysis, which included factor analysis and multiple regression, identified the strength attributed to each factor to the outcome explained by the PA. The last statistical assessment was performed using the machine-learning approach (ML). This regression-based statistical technique predicted the numerical value related to PA based on the inputs of the malnutrition indicators represented by different screening tools [35, 36]. The correlation coefficient along with error metrics (i.e. mean absolute error (MAE), root mean squared error (RMSE)) are produced by ML model to assess deviations from the actual assessment of PhA [18, 37]. ML regression was developed using the caret-learn model. Clinical parameters were used to develop the models using five-fold cross-validation in the Software-R with a BIA measurement represented by PhA. This statistical procedure was assisted by a professional statistician who built and validated the ML model.

## Results

### Demographic and admission characteristics

A total of 709 patients were assessed in the study observation. Patients’ characteristics on admission are presented in Table 1. As shown, the mean patients’ age was 51 years and the majority of these patients were male (65.9%). Patients were distributed over different medical diagnoses in which the majority (>70%) had initially been diagnosed on admission with sepsis (30.5%), followed by respiratory disorders (24.8%), and traumas (19.2%). The average BMI estimated on admission was 26.73 Kg/m^2^ which is considered within the overweight range. The admission mortality risk assessment represented by APACHE II and SOFA scores indicated moderate death risk (18.22 and 7.15, respectively). The median length of ICU stay was 12 days, while EN therapy was 7 days, and MV was 10 days. The number of reported deaths among the observed patients was 85 cases, representing a 12% mortality rate (Table 1).

**Table 1.** Admission and observation characteristics of the sample (N=709)

**Variable**	**Category**	**n**
**Age**, Mean (SD)		51.04 (12.57)
**Gender**, n (%)	Male	468 (65.9%)
	Female	241 (34.1%)

**Admission Diagnosis**, n (%)	Sepsis	216 (30.5%)
	Respiratory	176 (24.8%)
	Trauma	136 (19.2%)
	Cardiovascular	97 (13.7%)
	Neurologic	35 (4.9%)
	Gastrointestinal	22 (3.1%)
	Other	27 (3.8%)

**Admission BMI (Kg/m^2^)**, n (%)	Underweight (<18.5)	11 (1.6%)
	Normal (18.5−24.9)	272 (38.4%)
	Overweight (25−29.9)	260 (36.7%)
	Obese (>30)	166 (23.4%)

**Admission APACHE II score**, Mean (SD)		18.22 (6.5)
**Admission SOFA score,** Mean (SD)		7.15 (3.0)

**Length of ICU stay (Days)**, Median (IQR)		12 (10–19)
		Range: 6–61 days

**Duration of EN (Days)**, Median (IQR)		7 (4–10)
		Range: 1–30 days

**Duration of MV (Days)**, Median (IQR)		10 (5–13)
		Range: 1–36 days

**Mortality**, n (%)	Number of deaths (%)	85 (12.0%)
	Day of death, Median (IQR)	Day 8^th^ day (5^th^ – 11^th^)

N: Study sample, n: Frequency, SD: Standard Deviation, %: Percent, IQR: Inter-quartile range, ICU: Intensive care unit, EN: Enteral Nutrition, MV: Mechanical Ventilation, APACHE: Acute Physiologic Assessment and Chronic Health Evaluation, SOFA: Sequential Organ Failure Assessment.

### Outcomes of malnutrition screening in the critically ill

As mentioned, the nutritional screening carried out for the observed patients was articulated in different measures. Table 2 demonstrates the nutritional outcomes for all enetrally fed, mechanically ventilated patients at two stages: the admission stage and the assessment stage. As shown in the Table, the assessment of nutritional status using the BIA technology was conducted on the median 9^th^ day of admission (IQR: 7–11), whereas the other screening measures (MUST, NUTRIC, and NRS-2002) were obtained at the first 24 hours of admission. Regarding the admission stage, there was a convergence in the proportion of patients who fulfilled a low nutritional risk score using NUTRIC, MUST, and NRS-2002 tools (54.9%, 53.6%, and 46.4%, respectively). Likewise, the proportions of patients with a high risk for malnutrition were consistent across the three tools (45.1%, 46.4%, and 53.6%, respectively. At the assessment stage, more than 50% of patients demonstrated moderate to severe risk for malnutrition, while 44% demonstrated mild risk for malnutrition based on the PhA value classification. The segmental analysis of body compositions, from which PhA was derived, revealed low SMM and ECW/TBW ratio, whereas body fat mass was normal for both male and female patients (Table 2).

**Table 2.** Malnutrition screening using bioelectrical, physiological, and qualitative measures (N=709)

**Variable**	**Category**	**Value**
Assessment day of BIA since admission, Median (IQR)		Day 9th (7th – 11th)

Phase Angle (PA) score	>4 “Mild malnutrition”, n (%)	312 (44.0%)
	≤4 “Severe Malnutrition”, n (%)	397 (56.0%)
	PA mean (SD)	3.27 (1.58)

Skeletal Muscle Mass (SMM) ‘lbs’, Median (IQR)	DECREASE	30 (24–34)
Body Fat Mass ‘lbs’, Median (IQR)	NORMAL	17 (6–24)
ECW/TBW Ratio, Median (IQR)	Decreased	0.4 (0.36–0.44)

Admission NUTRIC score	0–4 “Low Risk”, n (%)	389 (54.9%)
	5–9 “High Risk”, n (%)	320 (45.1%)
	NUTRIC mean (SD)	4.73 (2.0)

Admission MUST score, n (%)	0 “Low Risk”	380 (53.6%)
	1 “Medium Risk”	202 (28.5%)
	≥2 “High Risk”	127 (17.9%)

Admission NRS-2002, n (%)	<3 Low Nutritional risk	329 (46.4%)
	≥3 High nutritional risk	380 (53.6%)

N: Study sample, n: Frequency, SD: Standard Deviation, %: Percent, BIA: Bioelectrical impedance analysis, ECW: Extra Cellular Water, TBW: Total Body Water, lbs: Pound, MUST: malnutrition universal screening tool, NRS: nutritional risk screening, NUTRIC: nutrition risk in critically ill.

### Predictive characteristics of different nutritional screening tools

To assess the predictive ability of the included nutritional screening tools, sensitivity, specificity, and accuracy were calculated, assuming that PhA is the reference indicator for malnutrition in this study. Based on the data presented in Table 3, it is apparent that both MUST and NRS-2002 scored higher sensitivity values compared to NUTRIC scores (77.6% and 76.8%, respectively). Specificity testing revealed similar inference, so that both tools revealed high specificity values (93.3%, and 90.7%, respectively). These findings were also reflected in the positive predictive value (PPV) and the negative predictive value (NPV) in addition to the accuracy testing (Table 3).

**Table 3.** Predictive characteristics of malnutrition screening tools (N=709)

**Variable**	**Sensitivity**	**Specificity**	**PPV**	**NPV**	**Accuracy**
NUTRIC score	59.4%	73.1%	73.8%	58.6%	56.1%
MUST score	77.6%	93.3%	93.6%	76.6%	70.4%
NRS-2002	76.8%	90.7%	91.3%	75.5%	66.1%

BMI: Body mass index, MUST: malnutrition universal screening tool, NRS: nutritional risk screening, NUTRIC: nutrition risk in critically ill, PPV: positive predictive value, NPV: negative predictive value. NUTRIC, MUST, and NRS-2002 were set as Diagnostic tests. Phase Angle was set as True/Outcome.

**Fig. 2.**
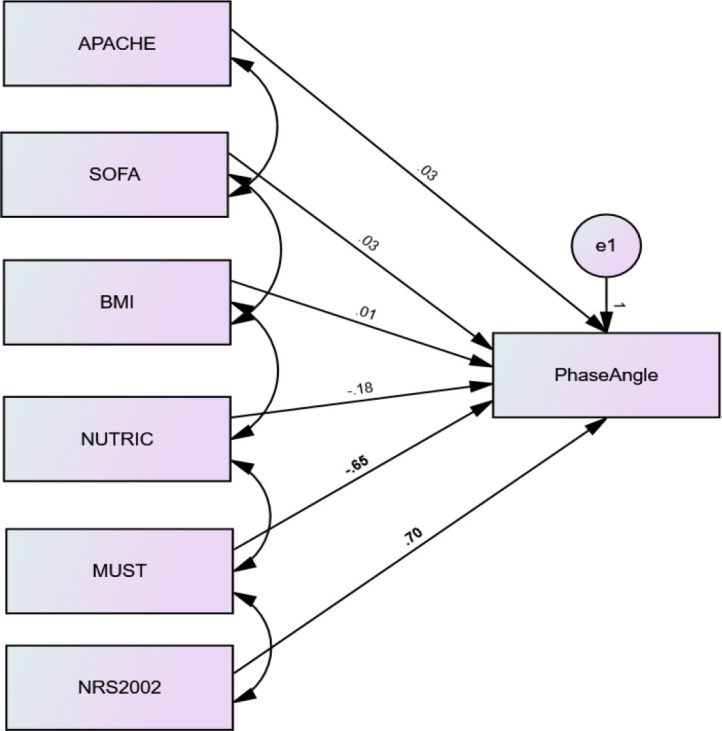
Structural equation model of relationship between observed variables (indicators) toward the PhA (dependent variable)

Further statistical evaluation was performed using a structural equation model which was created to examine the structural relationship between all observed variables (indicators) included in the study towards the PhA (dependent variable). As shown in Figure 2, the correlations measured in the model indicated that the highest correlations of estimates were scored by MUST and NRS-2002 scores (0.65, and 0.70, respectively). The rest of the indicators displayed low correlations to the estimation of the PhA values. This result affirmed the contribution of both MUST and NRS-2002 scores in predicting malnutrition in critically ill patients who were enterally fed and mechanically ventilated.

### ML approach to identifying effective nutritional screening tools

The application of ML approach provided additional evaluation of the nutritional screening tools assessed in this study. Focusing on the model presented in Table 4, a number of values represent the correlation coefficient (CC) and other error values. These error values explain the difference between the predicted scores from the regressing model and the actual score of the dependent variable (PhA). With respect to the correlation values in Table 4, it is evident that the highest correlations were exhibited in Model 5 and Model 6 (*r*= 0.90 and 0.91, respectively), which refer to MUST and NRS-2002 tools. The next strongest correlation was shown in NUTRIC tool, followed by APACHE II score and SOFA score. However, the evaluation of the model is unlikely to be informative without reading the error metrics which indicate the accuracy of prediction in addition to the amount of deviation from the actual scores. Accordingly, the lowest MAE, RMSE, RAE, and RRSE errors appeared in both Model 5 and Model 6. This is another confirmation that both MUST and NRS-2002 scores showed less deviation from the actual PhA scores, which in turn, support their predictability for malnutrition in the critically ill.

**Table 4.** Machine-learning model to identify effective nutritional screening tools (N=709)

**Models**	**CC**	**MAE**	**RMSE**	**RAE (%)**	**RRSE (%)**
Model 1	0.64	0.81	1.21	66.76	76.4
Model 2	0.54	0.87	1.33	71.41	84.39
Model 3	0.31	1.12	1.50	92.24	94.92
Model 4	0.69	0.70	1.15	57.60	72.76
Model 5	**0.90**	**0.28**	**0.71**	**23.45**	**45.13**
Model 6	**0.91**	0.29	0.66	24.27	42.03

CC: Correlation coefficient, MAE: Mean Absolute Error, RMSE: Root Mean Squared Error, RAE: The relative absolute error, RRSE: The root relative square error. Variables included in the models: Model 1 (APACHE II), Model 2 (SOFA), Model 3 (BMI), Model 4 (NUTRIC), Model 5 (MUST), Model 6 (NRS-2002).

It is noted that this model integrated the APACHE II score, SOFA score, and BMI score as the most significant influential factors in predicting the PhA. However, other clinical observation outcomes were not integrated to the model because they were insignificantly correlated with PhA values. These included the length of ICU stay and duration of EN duration. While the duration of MV significantly influenced the PhA in this study, it was not considered in the structural equation model because it was one of the SOFA score calculation elements.

Additionally, the Receiver-Operating Characteristic (ROC) curve was established to illustrate the performance of the binary PhA values at the selected threshold point of 4o in accordance with the MUST, NRS-2002, and NUTRIC scores. As shown in Figure 3, MUST was the most approximated performance to the reference line, followed by NRS-2002 and NUTRIC score, which showed the least approximation. MUST and NRS-2002 scored higher sensitivity (77.6%, and 76.8%, respectively) and specificity (93.3%, and 90.7%, respectively). Area Under the Curve (AUC), which was calculated based on the same threshold value of PhA, indicated that the MUST ability to discriminate malnutrition diagnosis was acceptable, followed by a moderate discrimination ability for NRS-2002, and NUTRIC (AUC= 0.85 [CI: 0.82−0.88], 0.69 [CI: 0.13−0.19], and 0.66 [CI: 0.65−0.73], respectively).

**Figure:**
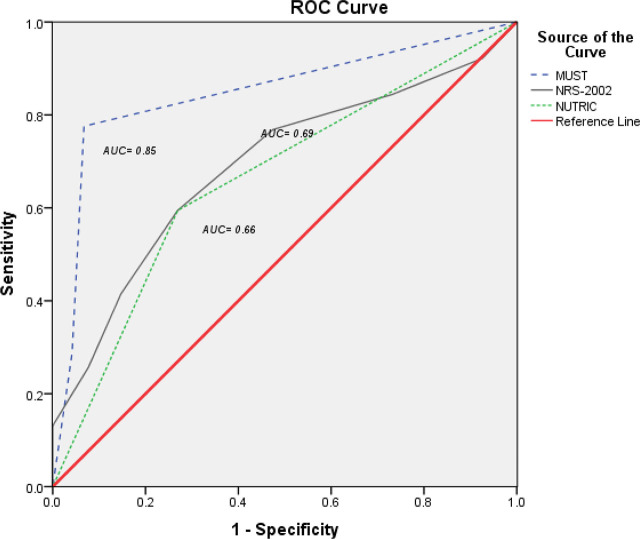


Considering the discriminative point of PhA at “4”, the three tools thresholds were inconsistent in estimating malnutrition risk, which was evidenced by the points that marked a highest sensitivity and specificity levels. For instance, MUST display a greater estimation of malnutrition risk at PhA 3.5, while NUS-2002 reported that at PhA score 2.5, and NUTRIC at PhA 2.2. However, this result means that MUST can be the most sensitive tool that predicts malnutrition risk even at less susceptible patients who manifest higher PhAs.

## Discussion

Recognizing the importance of nutritional assessment in critically ill patients who experience a significant energy deficit, is a core issue [38, 9]. The presence of coexisting factors such as the use of MV would exacerbate the problem [7]. Since evidence-based guidelines to deliver EN therapy safely, including the management of gastric residuals and aspiration reduction measures exist [4, 2], risk stratification is the key to minimizing the incidence of malnutrition and its related complications throughout ICU stay [15, 12]. In the absence of robust screening tools that cluster patients’ risk at admission, anticipating patients’ likelihood for malnutrition would not be easily achieved [39, 11].

Due to the heterogeneity of patients’ etiologies, some tools such as MUST, NUTRIC, and NRS-2002 were introduced as general screening tools that can be valid, especially for acutely hospitalized patients [15]. Special considerations were given in this study for enterally fed, mechanically ventilated patients because malnutrition is more likely to incident with MV [7]. Due to acute physiological deteriorations in these patients, acute physiological indicators, such as APACHE II and SOFA scores were integrated into patients’ assessment to determine their possible contribution to predicting malnutrition after a short period of stay in the ICU. In congruent with previous research [40, 41, 42], MUST and NRS-2002 scored high sensitivity and specificity in predicting malnutrition in intensive care. However, the NUTRIC score was viewed as less sensitive, specific, and accurate compared to the previous tools [28]. These results were also supported by the structural equation model which was created to identify the relationship between different variables with the BIA outcomes.

The validity of BIA technology has been tested for both cross-sectional and longitudinal in addition to test-retest reliability, which concluded that BIA technology showed high reliability, with very minimal accuracy error (0.0 to 0·49 %) [23, 21]. In a study that assessed the correlation between BIA and CT in 172 patients with malnutrition, CT scan and BIA were highly correlated in respect to both fat mass index and muscle mass index (0.79 and 0.83, respectively) [24].

It is argued that BIA may be affected by fluid shifts, edema, and measurement conditions in critically ill patients [24]. These alterations in the hydration status in the critically ill induce changes in membrane integrity as a result of fluid redistribution and shifting to the extracellular space [43, 21], something is observed in our study where the ECW/TBW ratio was decreased. While this condition may cause a reduction of PhA, measuring PhA at the earliest period of ICU admission is likely to minimize the confounding effect of altered hydration [44, 32].

Evidence obtained from machine-learning approach aimed at building a model assessing the predictive ability of each tool towards the actual PA value. Studies conducted in healthcare systems indicated that ML modeling can be an effective component of electronic health records to enhance earlier identification of patients at higher risk of malnutrition and develop management plans accordingly [36, 37]. A recent study conducted in neonatal ICU has developed ML algorithms to predict weight change at discharge using different demographic and clinical parameters. The study found that malnutrition detection after using ML algorithms becomes more efficient in terms of time and accuracy and impacts physicians’ performance and minimizes the risk of malnutrition [18]. In this study, the highest correlation coefficient and lower estimated errors addressed in the model indicate a strong predictive capacity for both MUST and NRS-2002 in predicting the risk of malnutrition among enterally fed, mechanically ventilated patients in the critically ill.

The predictive ability of certain screening protocols is extensively improved when a variety of subjective and objective measurements is integrated [13, 29, 1]. Developing an advanced modelling for estimating the risk of malnutrition at the earliest ICU admission can be well-established if utilized MUST and NRS-2002 along with adjunct BIA technology as possible.

### Limitations

Possible single-nation sample bias is acknowledged. Variations in EN practices between hospitals from different sectors were reported. For example, using intermittent versus continuous feeding methods, and using hospital-prepared versus pre-packed formulas. The study had not articulated the actual caloric delivery into patients’ observation to be assessed later for its correlation with BIA values. Potential device variability in BIA measurements is also recognized. For instance, controlling the effect of altered hydration on PhA vales would have provided more valid inferences.

## Conclusions

Early detection of malnutrition in the critically ill is still challenging for ICU professionals. Nutritional screening tools are key to rapid and early determination of malnutrition risk and should be coupled with proper nutritional assessment. Drawing attention to the significance of objective methods for nutritional assessment such as BIA, some subjective tools such as MUST and NRS-2002 showed their precision in predicting subsequent malnutrition risk for ICU patients from admission.
